# A Simplified Method for Analysis of Geosynthetic Reinforcement Used in Pile Supported Embankments

**DOI:** 10.1155/2014/273253

**Published:** 2014-08-19

**Authors:** Kang Fei

**Affiliations:** Institute of Geotechnical Engineering, Yangzhou University, Yangzhou 225009, China

## Abstract

The inclusion of geosynthetic reinforcement in the piled embankment can help transfer loads to the piles and reduce total and differential settlements. In order to select the appropriate reinforcement material, the reasonable calculation of the deflection and tension is very important. Current design methods usually do not represent the true three-dimensional (3D) nature of the displacements, strains, and stresses of the geosynthetics, and the resulting error may be large and cannot be neglected in some cases. In this study, two- and three-dimensional finite element analyses were conducted to identify the behavior of geosynthetic reinforcement and investigate the accuracy of the assumptions made in the current design methods. Based on the numerical results, a new 3D deflected shape of the geosynthetic reinforcement was suggested, and then the corresponding governing equation was derived and solved based on the membrane theory. To investigate the validity of the proposed method, the predicted maximum deflection, deflection shape, and the developed tensile force of the geosynthetics have been compared with the experimental data collected from the literatures and finite element analysis results.

## 1. Introduction

For the construction of embankments on very soft soils, certain techniques must be used in order to enhance stability and overcome the problem of settlement [1, 2]. One of these techniques is geosynthetic reinforced and pile supported (GRPS) embankment. Because this technique can save the time of construction and significantly improve performance in terms of displacements, GRPS embankment is increasingly used in China [3], Germany [4], The Netherlands, and other countries [5, 6].

In the GRPS embankment system, the differential settlement or shear deformation is caused in the embankment fill due to the presence of the soft foundation soil. The resultant shear stress increases the load applied on the piles and reduces the pressure on the geosynthetic reinforcement. This load transfer mechanism was termed as the soil arching effect by Terzaghi [7]. Sufficient studies have been performed to investigate the soil arching effect and the influencing factors including the column size and spacing and embankment height [8–12].

The remaining portion of the embankment load after soil arching effect is carried by both the geosynthetic reinforcement and the soft subsoil. Due to the fact that the geosynthetic reinforcement has negligible bending stiffness, it can only resist forces applied transversely to its longitudinal axis by a change in geometry. In other words, the geosynthetic reinforcement will be deflected and stretched. As a consequence, the vertical components of the developed tensile forces will transfer part of the load to the piles and reduce the load carried by the soft soil. This load transfer mechanism was known as the tensioned membrane effect [13, 14]. In order to select the appropriate reinforcement material, the tensile force in the geosynthetics must be calculated at first. A number of studies have been undertaken by various researchers to investigate the behavior of the geosynthetic reinforcement and to determine a design method [15–19]. Almost all the current methods adopt the similar procedure. First, the deformed shape of geosynthetic reinforcement is assumed as a smooth curve. Second, the vertical displacement, strain, and tension in the geosynthetics are determined by solving the governing equations based on the assumed deformed shape in order to satisfy the static equilibrium of the system. In John's [15] and Rogbeck et al.'s [16] methods for plane strain condition, the load acting on the reinforcement was assumed to be vertical and uniformly distributed along the deflected length, and the resultant deflected shape of the geosynthetic reinforcement was catenary. For ease of computation, British Standard BS8006 [18] assumed the vertical load to be distributed uniformly along the horizontal span of the reinforcement between two adjacent pile caps, which results in a parabolic shaped deflection. Low et al. [8] idealized the deformed shape of the geosynthetic reinforcement as a circular arc, which implies that the stresses are normal to the geosynthetics. The circular arc assumption was also adopted by Giroud et al. [13] when designing geosynthetic layer over an infinitely long void.

For plane strain problem, all the three deformed shape models were found to give reasonable results of maximum vertical deflections, tension strains, and tension forces [20]. However, these simplified models do not represent the true three-dimensional nature of the displacements, strains, and stresses of the geosynthetic reinforcement in piled embankment. The reliability of the current design method and the deformation feature for 3D problem need to be further studied.

In this study, finite element analyses were conducted using ABAQUS to identify the behavior of geosynthetic reinforcement and investigate the accuracy of the assumptions made in the current design methods. Based on the results, a new simplified analytical method was proposed and presented. To investigate the validity of the proposed method, the predicted results have been compared with experimental data collected from the literatures and finite element analysis results.

## 2. 2D Finite Element Analysis

### 2.1. Analysis Model

**Figure 1:**
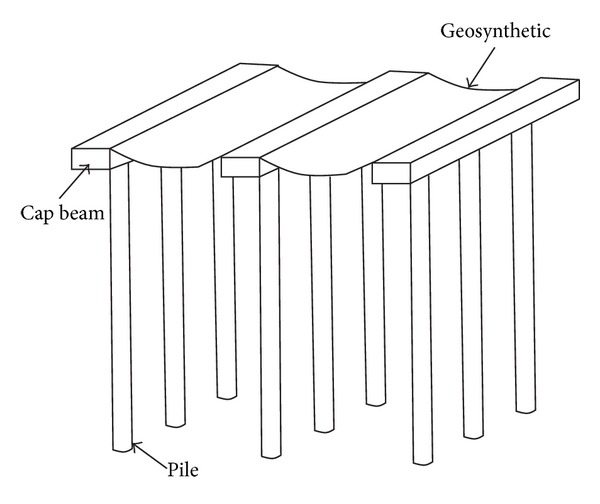
Embankment piles with cap beams and geosynthetic reinforcement.

The two-dimensional model was selected based on a typical piled embankment system using cap beams and geosynthetics. As shown in Figure 1, the precast concrete cap beams are placed perpendicularly to the longitudinal axis of the embankment, and one layer of geosynthetic reinforcement is placed on the top of the cap beams to enhance load transfer and reduce differential settlement. To simplify the problem and focus on the deformed shape of the geosynthetic reinforcement, the soil response is uncoupled from the geosynthetic reinforcement. Considering the purpose of the analysis which is to establish a relationship between the applied pressure, the deflection, the tension stress, and the strain of the geosynthetic, five different magnitudes of pressures, that is, 10, 20, 30, 40, and 50 kPa, were adopted in the analyses. In addition, the applied pressure was assumed to be uniform and vertical during the analysis, and no support from the subsoil is accounted.

**Figure 2:**
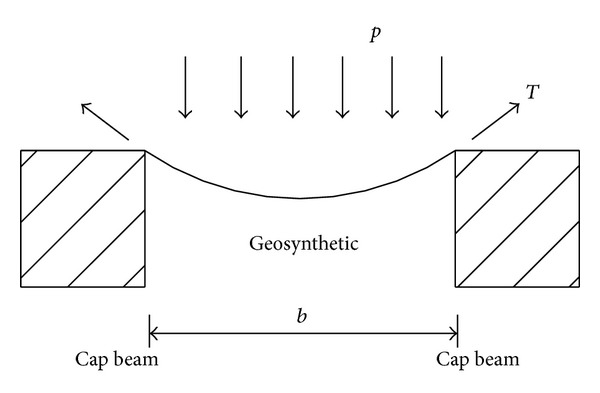
Sketch of 2D model.

Because the piles are relatively rigid compared with the foundation soil between the piles, the geosynthetic reinforcement is assumed to be fixed at the edge of the cap beams. The net spacing between the piles, that is, the initial length of the geosynthetic reinforcement, is *b* = 1.5 m (Figure 2). A series of cable elements that have no flexural rigidity and could resist tension only was used to model the geosynthetic reinforcement. The behavior of the geosynthetic reinforcement is assumed to be linear elastic and the tensile stiffness of the geosynthetic is *K*_*g*_ = 1500 kN/m.

Verification analyses have been performed to gain an understanding of the appropriate numerical modeling procedures and required mesh refinement for the problem. In all of the analyses, the geometrical nonlinear effect is included to model the tensioned membrane effect appropriately.

### 2.2. Analysis Results

#### 2.2.1. Geosynthetic Deflection

In this section, the results of finite element analyses are compared with Giroud's method and BS8006 to investigate the accuracy of the assumptions of the deflected geosynthetic shape. For ease of comparison, both methods are introduced briefly as follows.

Giroud et al. [13] idealized the deformed shape of the geosynthetic reinforcement as a circular arc, the strain *ε*, is given by

(1) $$\begin{array}{c} \varepsilon =2\Omega \text{si}{\text{n}}^{-1}\left[\frac{1}{\left(2\Omega \right)}\right]-1, \end{array}$$

where *Ω* is a dimensionless factor given by

(2) $$\begin{array}{c} \Omega =\frac{1}{4}\left[\frac{2w_{\max}}{b}+\frac{b}{2w_{\max}}\right], \end{array}$$

where *w*_max⁡_ is the maximum deflection of the geosynthetic reinforcement. The tension in the geosynthetic reinforcement *T* is computed as follows:

(3) $$\begin{array}{c} T=pb\Omega . \end{array}$$

In the BS8006 method [18], the deflected shape of the geosynthetic reinforcement is approximated as a parabola, and the strain and tension are computed using the following:

(4) ε=8wmax⁡23s−a2,

(5) T=WTs−a2a1+16ε,

where *W*_*T*_ is the distributed load per unit length, for plane strain condition, *W*_*T*_ = *pa*; *a* is the width of the pile cap; *s* is the central spacing between piles, and the other notations are as defined before.

In practice, the maximum deflections in (2) and (4) are determined by trial and error to ensure that the calculated tension and strain satisfy the following:

(6) $$\begin{array}{c} T=K_{g}\varepsilon . \end{array}$$

**Figure 3:**
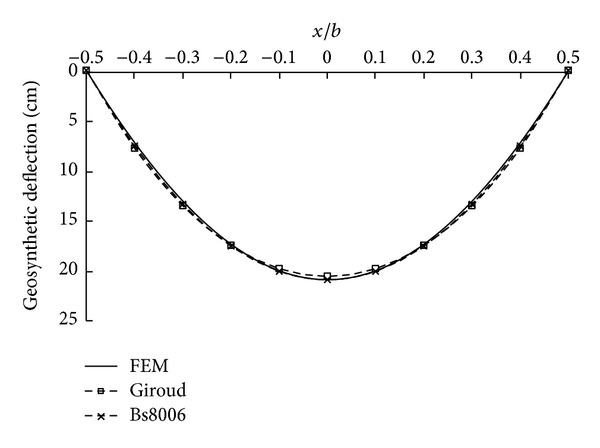
Comparison of geosynthetic deflections computed by 2D FEM and design methods (*p* = 50 kPa, 2D).

The computed shapes of the deflected geosynthetic reinforcement at the pressure of 50 kPa were shown in Figure 3. The notation *x*/*b* in the figure means the distance to the midpoint of the geosynthetic reinforcement normalized by the clear span *b*. It can be seen that the deflected shape of the geosynthetic reinforcement can be adequately described by both the parabolic and circular geometries. The maximum deflections computed by different methods are 20.5 cm (Giroud's method), 20.9 cm (BS8006), and 20.8 cm (FEM), respectively. The discrepancy between the current simplified design methods and FEM result was found to be less than 2%.

#### 2.2.2. Geosynthetic Tension

**Figure 4:**
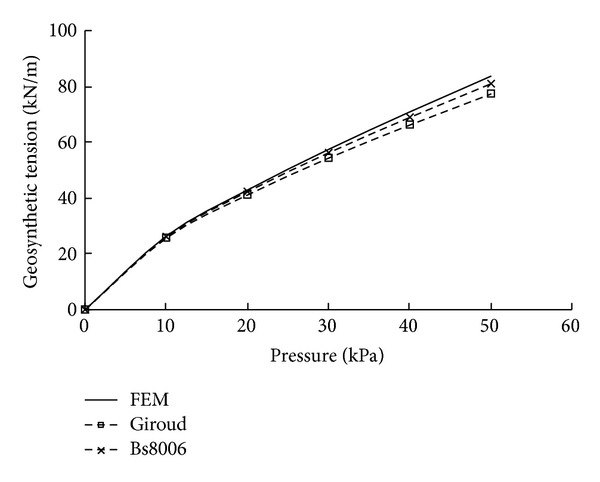
Comparison of geosynthetic tensions computed by 2D FEM and design methods (2D).

Figure 4 compares the predicted relationships between the tension and the applied pressure. It can be observed that there is a slightly obvious difference in the predicted tensions, though the computed deflected shapes of different methods are very close. The tension estimated from FEM analysis is approximately 1.08 and 1.04 times that calculated assuming a circular and parabolic deflected shape, respectively. It should be noticed that the load acting on the geosynthetic was assumed to be vertical and distributed uniformly in the finite element analysis, so the determined deflected shape is also close to a parabola, so the predicted tension is almost identical to that calculated from BS8006.

## 3. 3D Analysis

### 3.1. Analysis Model

Because the current design methods do not represent the true 3D deformation nature of the geosynthetic reinforcement, 3D finite element analyses were conducted to get a better understanding of the deflected shape and the corresponding tensile forces developed in the geosynthetic reinforcement.

**Figure 5:**
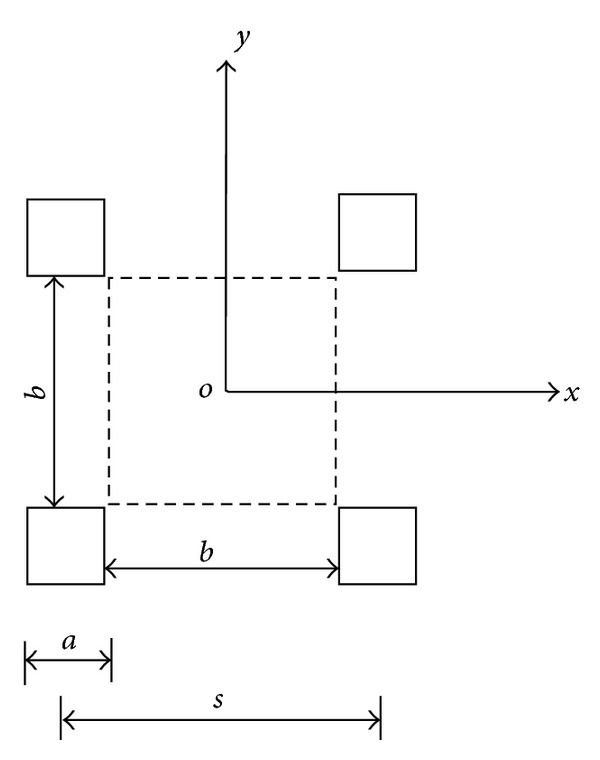
Sketch of 3D model.

The 3D model was selected based on a typical piled embankment system using individual caps and geosynthetics. As shown in Figure 5, the piles were arranged in a square pattern with a cap width of *a* = 0.5 m. In the analyses, three net spacings (*b* = 1.0 m, 1.5 m, and 2.0 m) and five pressures (*p* = 10, 20, 30, 40, and 50 kPa) were adopted. The tensile stiffness of the geosynthetic was *K*_*g*_ = 1500 kN/m.

To simplify the problem, only one unit cell of the geosynthetic reinforcement was modeled. Similar to the 2D analysis, the load above the geosynthetic reinforcement was assumed to be uniform and vertical, and the geosynthetic reinforcement was assumed to be fixed at all 3 directions at the pile edges. The shell elements with small bending stiffness were used to discretize the geosynthetic.

### 3.2. Analysis Results

#### 3.2.1. Geosynthetic Deflection

**Figure 6:**
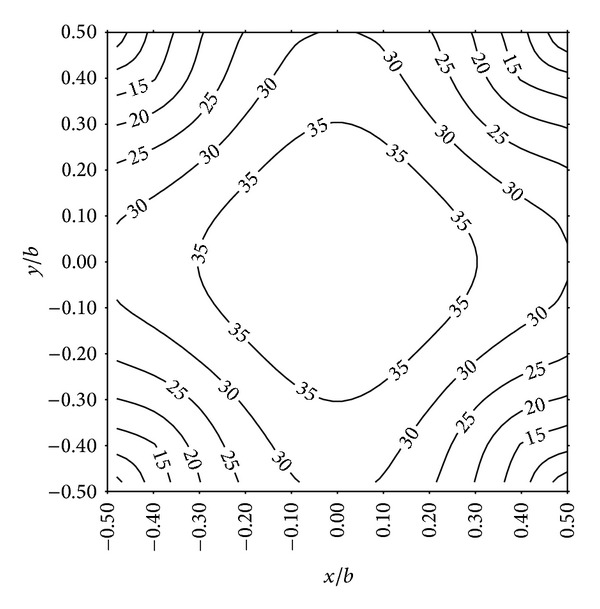
Computed geosynthetic deflection (*p* = 50 kPa, *b* = 1.5 m, 3D).

**Figure 7:**
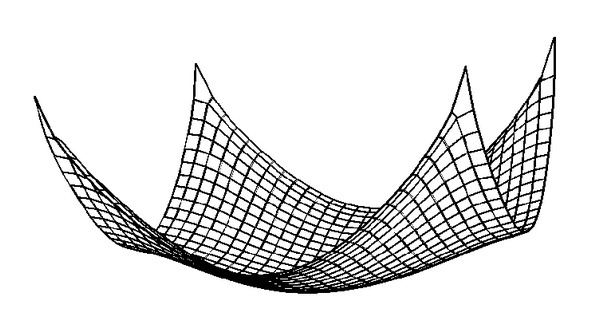
Sketch of three-dimensional deformed shape of the geosynthetic reinforcement.

The contour of computed geosynthetic deflection under a pressure of 50 kPa and a clear spacing of 1.5 m is plotted in Figure 6. For clarity, Figure 7 gives the sketch of the 3D deformed shape. It can be seen that the downward deformation increases very sharply near the pile edges, while the change of the deflection is small at the central zone of the geosynthetic reinforcement. As a result, the strain in geosynthetic reinforcement is not uniformly distributed as assumed in the current 2D design methods. The maximum deflection at the center of the geosynthetic reinforcement is 39.7 cm, which is significantly higher than 20.9 cm obtained from the 2D analysis. Consequently, the tension generated in the geosynthetic reinforcement is underestimated if the 3D nature of the problem is ignored, so a 3D model is needed to be developed, which will be introduced in the following sections.

#### 3.2.2. Geosynthetic Tension

**Figure 8:**
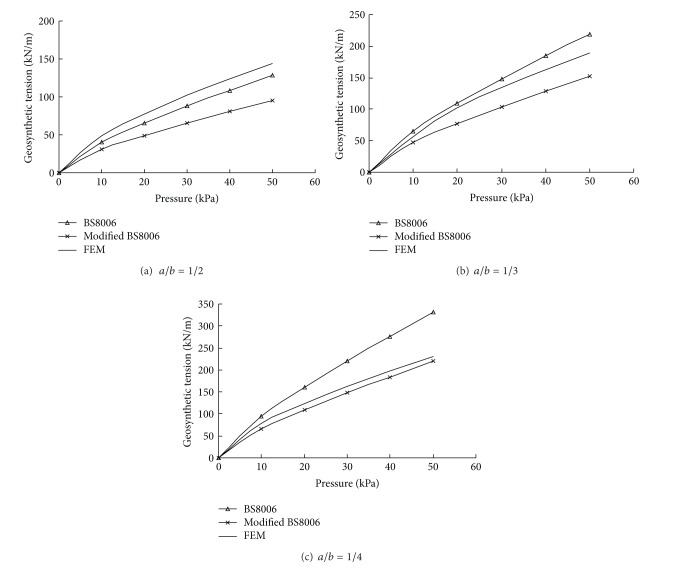
Geosynthetic tension versus pressure (3D).

**Figure 9:**
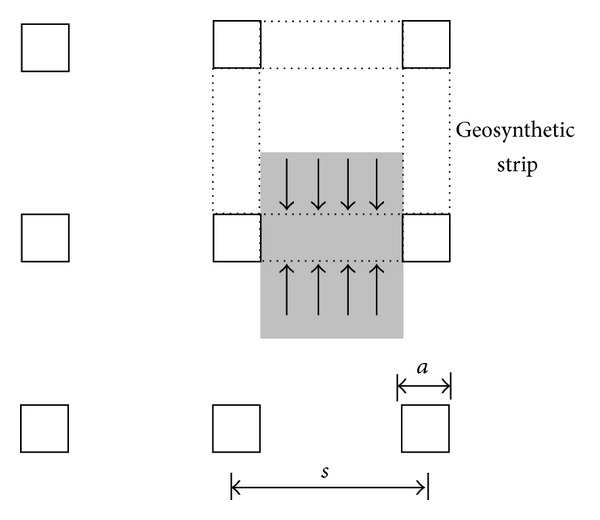
Calculation of the load to the geosynthetic strips in BS8006 (after [21]).

**Figure 10:**
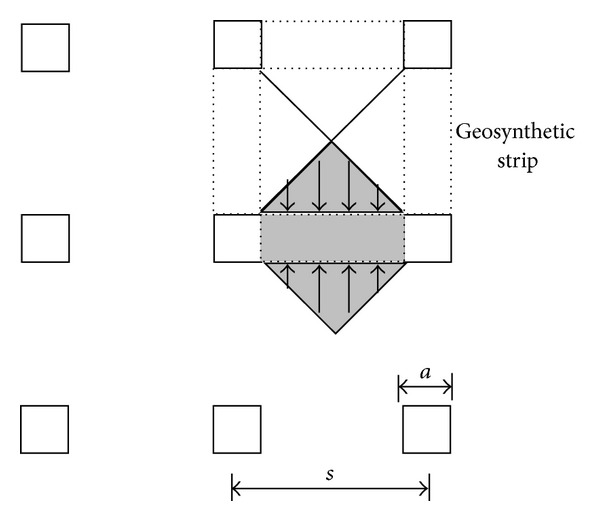
Calculation of the load to the geosynthetic strips in modified BS8006 (after [21]).

The tensions computed at the centroid of the element adjacent to the pile edge are compared with the results of BS8006 in Figure 8. To account the 3D configuration of the GRPS embankment approximately, BS8006 recommends the line vertical load *W*_*T*_ used in (5) which is calculated by multiplying average vertical stress by the unsupported area (shade area as shown in Figure 9) that transfers the tensile loads onto the reinforcement strip:

(7) $$\begin{array}{c} W_{T}=\frac{p\left(s-a\right)s}{\left(s-a\right)}=ps. \end{array}$$

van Eekelen et al. [21] pointed that BS8006 overestimated the load on the reinforcement by applying line load to directions both along and perpendicular to the road axis. In other words, the load on the geosynthetic is included twice. They suggested a modified method to calculate *W*_*T*_ (Figure 10):

(8) $$\begin{array}{c} W_{T}=\frac{p\left(s^{2}-a^{2}\right)}{2\left(s-a\right)}=\frac{p\left(s+a\right)}{2}. \end{array}$$

Once the line load *W*_*T*_ is determined, the tension in the geosynthetic reinforcement is also calculated based on (5). The corresponding result is indicated as modified BS8006 in Figure 8.

As expected, the tensions given by BS8006 were much higher than the modified BS8006 as the line load *W*_*T*_ was enlarged. For the case of *s* = 2.0 m and *a* = 0.5 m, the load is overestimated by 60%, and thus the corresponding reinforcement tension is about 45% higher. Though the reinforcement load of Modified BS8006 is more reasonable than that in BS8006, the tension is underestimated relative to the finite element analysis. For *a*/*b* = 1/3, the modified BS8006 geosynthetic tensions were approximately 35% lower than that of the 3D finite element analysis. The most possible reason is that the geosynthetic drops sharply at the edges of the pile caps, so the resultant strains and stress are particularly large near the corners. The methods that use a cable model connecting two adjacent piles without consideration of three-dimensional effects do not address such strain and stress concentrations. This also explains why the geosynthetic tension calculated by BS8006 was underestimated for *a*/*b* = 1/2 even if the load is overestimated.

## 4. Proposed Simplified Method

### 4.1. Assumption

The behavior of the geosynthetic reinforcement is assumed to be linear elastic and be in tension only. The pressure applied on the geosynthetic reinforcement is uniform and vertical. In addition, it is also assumed that the piles are arranged in a square pattern and no support from the subsoil is accounted.

Based on the finite element results, a new function is used to model the three-dimensional deformed shape of the geosynthetic:

(9) $$\begin{array}{c} w=A\left(x^{2}+y^{2}-\frac{b^{2}}{2}\right)+B\left(x^{2}y^{2}-\frac{b^{4}}{16}\right), \end{array}$$

where *A* and *B* are coefficients to be determined.

### 4.2. Development and Solution of the Governing Equation

Ignoring the deformation at the *x* and *y* directions, the strain component along *x* direction in plane coordinates is given by

(10) $$\begin{array}{c} \varepsilon_{x}=\sqrt{1+{\left(\frac{\partial w}{\partial x}\right)}^{2}}-1. \end{array}$$

Though the deflection is large, the strain components still remain small compared with the unity. Thus *ε*_*x*_ can be approximated as

(11) $$\begin{array}{c} \varepsilon_{x}=\frac{1}{2}{\left(\frac{\partial w}{\partial x}\right)}^{2}=2\left(A^{2}x^{2}+2ABx^{2}y^{2}+B^{2}x^{2}y^{4}\right). \end{array}$$

Similarly, the strain components *ε*_*y*_ and *γ*_*xy*_ are given by

(12) εy12∂w∂y2=2A2y2+2ABx2y2+B2x4y2,γxy∂w∂x∂w∂y=4Ax+Bxy2Ay+Bx2y.

The constitutive relations between the membrane forces *N*_*x*_, *N*_*y*_, and *N*_*xy*_ and the strains *ε*_*x*_, *ε*_*y*_, and *γ*_*xy*_ are

(13) Nx=Et1−ν2εx+νεy,Nx=Et1−ν2εy+νεx,Nxy=Et21+νγxy,

where *E* and *v* are the modulus of the elasticity and Poisson's ratio, respectively, and *t* is the thickness of the membrane.

Based on the elastic membrane theory, the equilibrium equation along the vertical direction is

(14) $$\begin{array}{c} N_{x}\frac{\partial^{2}w}{\partial x^{2}}+N_{y}\frac{\partial^{2}w}{\partial y^{2}}+2N_{xy}\frac{\partial^{2}w}{\partial x\partial y}+p=0. \end{array}$$

By integrating the equilibrium equation along the diagonal line (*x* = *y*) and the edge (*y* = 0), we obtain

(15) 1+ν3b3A3+5+ν20b5A2B+7−ν112b7AB2 +3−ν576b9B3+1−ν2Etpb=0,1+ν6b3A3+ν40b5A2B+1−ν2Etpb=0.

Equations (15) can be solved by the Newton-Raphson method. Once the variables *A* and *B* are calculated, the deflection of the membrane, the corresponding strains, and the membrane forces can be determined.

It should be noticed that the suggested function is an approximation to the true 3D deformation shape, and because only the vertical deflection is accounted, the solution is approximate. However, it is believed that the proposed method can significantly simplify the calculation and yield accurate enough results.

## 5. Validation of the Simplified Analytical Method

### 5.1. Comparison with Model Tests of Ballooning Membrane

A detailed set of model tests carried out on four corner points constrained square membrane was described by Shi and Burnett [22]. The purpose of the model tests was to measure the deflections of the membrane under different air pressures. The thickness of the membrane used in the experiments was 0.16 mm, with the dimension of 406 mm × 406 mm. The material properties of the membrane were Yang's modulus of *E* = 875 MPa and Poisson's ratio of *v* = 0.24. Four sets of pressures, that is, *p* = 25, 50, 100, and 150 Pa, were perpendicularly applied on the membrane. The deflection of the ballooning membrane was measured at eight evenly distributed points over half span of both the side and the diagonal.

**Figure 11:**
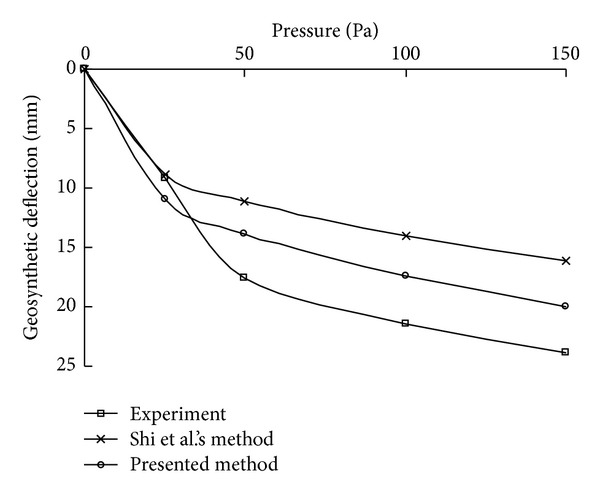
Comparison of the maximum deflections of the membrane under different pressures.

**Figure 12:**
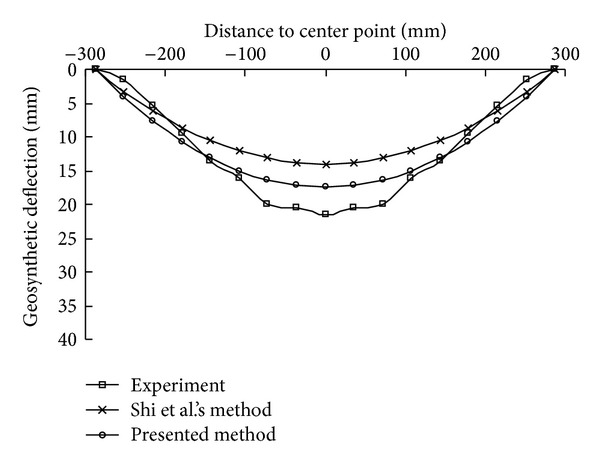
Comparison of deflections along the diagonal.

**Figure 13:**
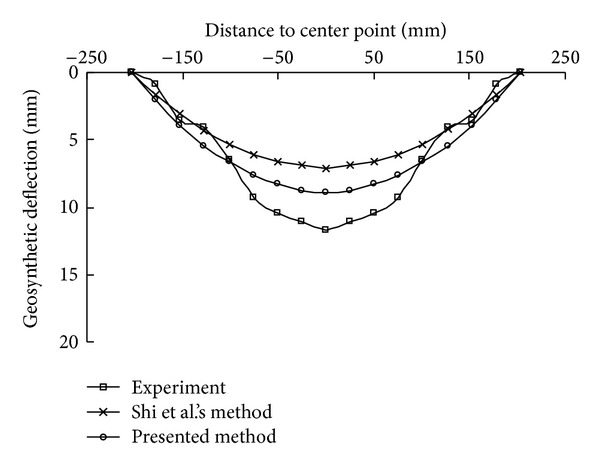
Comparison of deflections along the edge.

The maximum deflections obtained from the experiments, Shi's method, and the proposed method are shown in Figure 11. As can be seen, the nonlinear trend of increase of the maximum deflection with the applied load is modeled well by the proposed simplified analytical method, and the predicted maximum deflections are closer to the experimental values than those of the original method, though there are still some differences. The deflections along the diagonal and the edge under a pressure of 100 Pa are plotted in Figures 12 and 13, respectively. The results also show that the assumed shape function in the presented method is appropriate.

### 5.2. Comparison with Model Tests of GRPS Embankment

A series of three-dimensional model tests of GRPS embankment were carried out by Demerdash [23] to investigate the effects of soil arching and tension membrane. The physical model was designed to represent a square grid of individually capped piles centrally located within an embankment. A movable base supported on hydraulically operated jacks was used to model the soft ground. The widths of the three pile caps investigated were 200 mm, 250 mm, and 300 mm, respectively, and the centre line spacing between the pile caps was maintained unchanged at 600 mm for all tests. Two kinds of the geotextiles were used in the tests, and the stiffness moduli were approximately 165 and 500 kN/m, respectively. The deflection of the geotextile mesh was monitored along the diagonal and the edge using the draw wire transducers.

**Figure 14:**
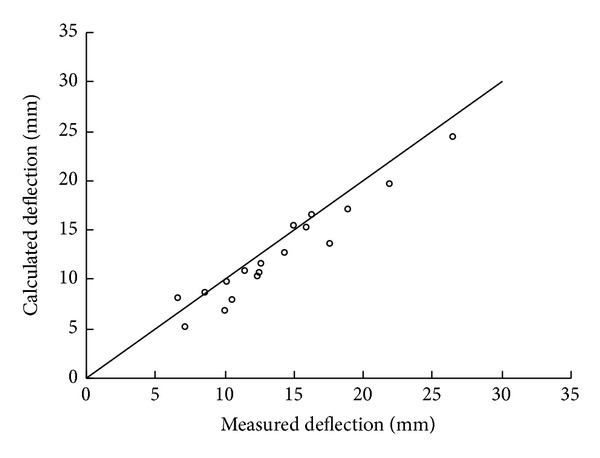
Experimental versus calculated deflections.

Because the tension of the geotextile was not measured in the test, only the deflection results of the model tests were compared with the predictions by the proposed method. The measured deflection at the center of the geotextile was used to calculate the deflections at the midpoint of the edge, the quarter-span deflections of the diagonal, and the edge based on the proposed 3D deformation shape.  Figure 14 shows the experimental versus the calculated deflections at different locations. The 45° line superimposed in the figure represents a perfect fit relationship between the experimental and calculated values. As can be seen, the calculated deflections based on the proposed shape function are in good agreement with the experimental values.

### 5.3. Comparison with Finite Element Analyses

To verify the accuracy of the predicted geosynthetic tension by the proposed simplified analytical method, the results were compared with those of finite element analyses given in Section 3.2.2.

**Figure 15:**
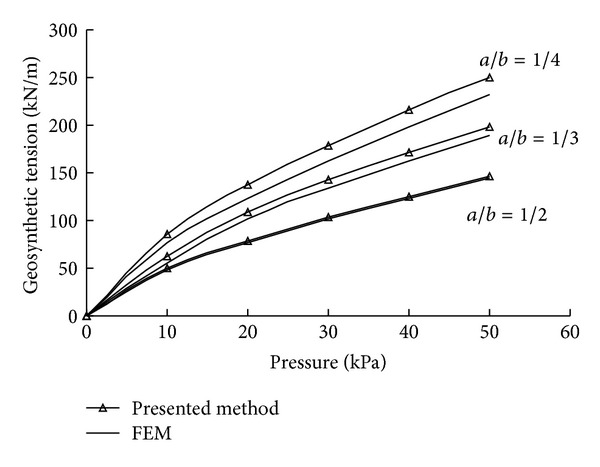
Comparison of geosynthetic tensions calculated by FEM and presented method.

Figure 15 compares the geosynthetic tensions calculated by FEM and presented the method at different ratios of the pile caps' width to the clear spacing *a*/*b*. As can be seen, the results from the proposed simplified analytical method agree well with those from the finite element analyses. The discrepancy between the calculated values of these two approaches was found to be less than 10%. In addition, the variations of the results of FEM and the proposed method follow the same trend, the tension of geosynthetic increases with increasing applied load. It is also clear that tension of geosynthetic decreases with increasing *a*/*b* as the portion of the load carried by geosynthetic becomes smaller.

## 6. Conclusions

To investigate the reliability of the current simplified methods for design of geosynthetic reinforcement in piled embankment, two- and three-dimensional finite element analyses were conducted at first. It was found that for two-dimensional problem, the deflected shape of the geosynthetic can be adequately described by both the parabolic and circular arc geometries. The discrepancy between the predicted geosynthetic tensions of current simplified design methods and finite element method was also very small. For three-dimensional problem, a marked difference was found among the geosynthetic tensions calculated using BS8006, modified BS8006, and finite element method since the true three-dimensional nature of the problem was not represented reasonably.

Based on the numerical results, a new deformation model was suggested to model the three-dimensional deflected shape of the geosynthetic reinforcement, and then the corresponding governing equation was derived and solved based on the membrane theory. By comparing the predicted maximum deflection, deflection shape, and the developed tensile force of the geosynthetic with those of model tests and finite element analyses, the simplified method proposed was verified to be convenient and accurate.
